# Severe alcohol use and COVID-19: implications for physical and mental health

**DOI:** 10.3389/fpsyt.2025.1640207

**Published:** 2025-11-05

**Authors:** Javier Calleja-Conde, Víctor Echeverry-Alzate, Sara Sánchez-Diez, Elena Giné, Kora-Mareen Bühler

**Affiliations:** ^1^ Cardenal Cisneros Higher Education Center, Madrid, Spain; ^2^ Department of Psychobiology and Methodology in Behavioral Sciences, Faculty of Psychology, Complutense University of Madrid, Madrid, Spain; ^3^ Neurociencia Básica y Clínica (NBC) Group, School of Life and Nature Sciences, Nebrija University, Madrid, Spain; ^4^ Department of Cell Biology and Histology, Faculty of Medicine, Complutense University of Madrid, Madrid, Spain

**Keywords:** COVID-19, alcohol, immunity, neuroinflammation, mental health, Long COVID

## Abstract

The COVID-19 pandemic has revealed and intensified the vulnerability of individuals with pre-existing medical and behavioral conditions, notably those related to substance use. Among these, chronic alcohol consumption represents a clinically significant, yet often under-addressed, vulnerability factor that may exacerbate both the acute severity and long-term consequences of SARS-CoV-2 infection. This narrative review examines the biological and clinical intersections between alcohol use and COVID-19, focusing on shared mechanisms of immune dysfunction, neuroinflammation, and disruption of the gut–brain axis. We synthesize current findings showing that both conditions compromise innate and adaptive immune responses, alter cytokine signaling, and weaken mucosal and blood–brain barriers. These changes contribute to cognitive and emotional dysregulation and may increase the risk of persistent neuropsychiatric symptoms, including those observed in Long COVID. In addition, we discuss how chronic alcohol use may alter host susceptibility to infection and affect the immune response to vaccination, with implications for treatment outcomes and recovery. Our findings highlight the need to integrate alcohol use disorder into COVID-19 risk assessments, clinical management, and long-term mental health care planning. A multidisciplinary approach is essential to address the overlapping biological pathways that link alcohol-related vulnerability to COVID-19 outcomes.

## 1 Introduction

The COVID-19 pandemic has profoundly impacted global health, not only through direct viral effects but also by exposing and amplifying pre-existing vulnerabilities. Among these, alcohol use disorders (AUD) represent a critical factor influencing both physical and mental health outcomes. This review aims to explore the biological interplay between severe alcohol consumption and SARS-CoV-2 infection, with a particular emphasis on how both factors interact through immune system dysfunction and ultimately converge in their impact on mental health.

While existing literature has separately addressed alcohol’s effects on immunity and mental disorders, and the neuropsychiatric consequences of COVID-19, few works have examined how these domains intersect. This review begins with a brief overview of SARS-CoV-2 and the clinical features of COVID-19, followed by contextual information on alcohol consumption patterns during the pandemic. The subsequent sections examine how both alcohol use and SARS-CoV-2 infection independently alter immune function and contribute to mental health vulnerability. The final section presents an integrated analysis of their combined impact, highlighting shared biological pathways and their implications for physical and psychological health.

## 2 Biological characteristics, transmission, and clinical profile of SARS-CoV-2

COVID-19 was first identified in late 2019 and was officially declared a global pandemic by the World Health Organization (WHO) on March 11, 2020 (1). Since its emergence, the disease has spread worldwide, with infection rates varying across regions and demographic groups. As of early 2025, the total number of reported cases has exceeded 777 million, with approximately seven million deaths (2). Despite extensive vaccination efforts that have significantly reduced infection rates and severe cases, the virus remains in circulation. In the first months of 2025, around 22,000 new cases were reported globally (2).

**Figure 1:**
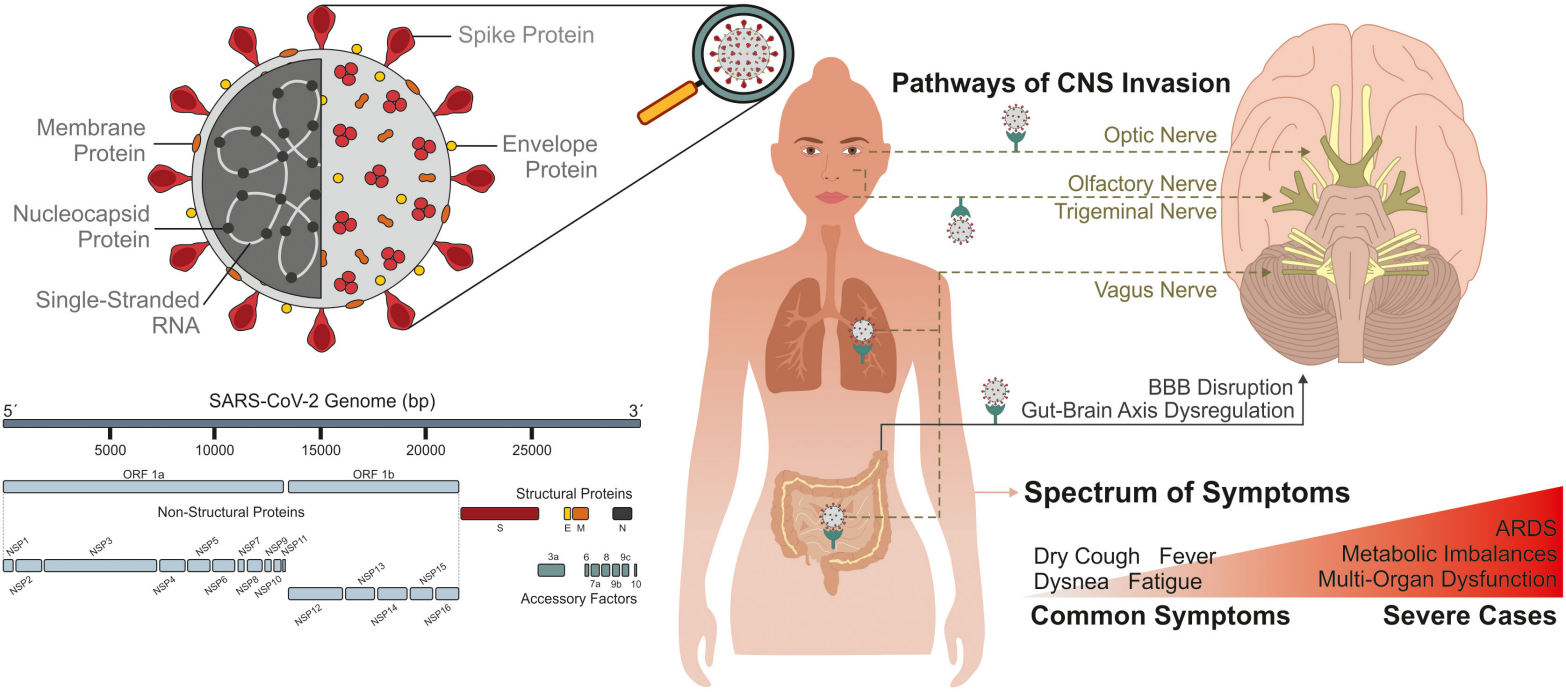
General characterization of SARS-CoV-2. Left: Structure of SARS-CoV-2 capsid (above) and genome components (below). The genomic structure of coronaviruses is highly conserved and includes three main regions. Open reading frames (ORFs) 1a and 1b contain two polyproteins that encode the nonstructural proteins (nsp). The last third of the genome encodes structural proteins, including the spike (S), envelope (E), membrane (M), and nucleocapsid (N) proteins. Accessory genes are interspersed throughout the genome. Right: CNS invasion Pathways of the virus and symptoms. The S protein is particularly critical for viral entry, as it binds to the angiotensin-converting enzyme 2 (ACE2) receptor present on host epithelial and endothelial cells, including those in the respiratory tract- primary route of infection-, gastrointestinal and endocrine systems, as well as peripheral and cranial nerves, and may reach the central nervous system by disrupting the blood–brain barrier (BBB). COVID-19 presents a wide spectrum of symptoms, ranging from mild to severe cases. This broad range of clinical manifestations observed in COVID-19 could be explained by the wide distribution of ACE2 receptors across diverse tissues and organs. ARDS: acute respiratory distress syndrome.

SARS-CoV-2, the virus responsible for the COVID-19 pandemic, belongs to the coronavirus family, which includes other significant pathogens such as SARS-CoV and MERS-CoV. While SARS-CoV-2 shares typical structural features with other coronaviruses (3), it exhibits distinct structural modifications that enhance its infectious potential (4). Structurally, SARS-CoV-2 is an enveloped virus with a positive-sense, single-stranded RNA genome approximately 30,000 nucleotides long. It encodes several structural proteins, including the spike (S), envelope (E), membrane (M), and nucleocapsid (N) proteins (**Figure 1**). The virulence, replication, and structure of SARS-CoV-2 are all dependent on these proteins (5). The S, E, and M proteins are present on virion membrane surfaces, while the N protein is involved in the binding and packing of the RNA genome. Among these proteins, the S protein is particularly critical for viral entry, as it binds to the angiotensin-converting enzyme 2 (ACE2) receptor present on host epithelial and endothelial cells, including those in the respiratory tract (6, 7). The distribution of this receptor significantly influences the clinical manifestations of COVID-19. In addition, a varying number of accessory proteins and non-structural proteins (NSPs) genes are interspersed between the structural genes. Accessory proteins, including ORF3a, ORF6, ORF7a, ORF7b, ORF8, and ORF10 proteins, are not essential for virus replication but play pivotal roles in immune evasion and pathogenesis (8, 9). NSPs, on the other hand, are crucial for the virus life cycle. Among these, one of the most important non-structural proteins is Nsp5, also known as the main protease (Mpro). It is essential for viral replication as it cleaves polyproteins translated from the viral RNA, producing functional proteins necessary for replication (10).

SARS-CoV-2 is primarily transmitted via infectious respiratory fluids, with transmission occurring even during the incubation period and from asymptomatic or presymptomatic individuals—factors that have significantly contributed to the global spread of COVID-19 (11, 12). SARS-CoV-2 spreads through airborne transmission via aerosolized particles that can remain suspended, particularly in poorly ventilated environments, and through larger respiratory droplets expelled during coughing, sneezing, or talking (12, 13). Although surface transmission (fomites) is possible, current evidence suggests it plays a limited role. Ocular exposure via the conjunctival epithelium also represents a possible entry route (14, 15), as well as fecal-oral transmission; some cases of gastrointestinal infections without respiratory symptoms have been reported (16). Vaccination has been shown to reduce viral transmission (17).

SARS-CoV-2 primarily enters the human body via the respiratory tract; however, increasing evidence indicates that the virus can also exploit alternative entry routes, affecting multiple organ systems. Beyond the pulmonary epithelium, viral particles have been detected in the gastrointestinal and endocrine systems, likely due to high expression of viral entry receptors in these tissues (18). Furthermore, SARS-CoV-2 has demonstrated neuroinvasive potential, possibly reaching the central nervous system (CNS) either through retrograde axonal transport via peripheral nerves or by compromising the integrity of the blood-brain barrier (BBB) (19, 20) (**Figure 1**). Entry is mediated by the viral spike (S) protein, which binds to ACE2 receptors and is facilitated by host proteases such as transmembrane serine protease 2 (TMPRSS2) and cathepsin L (19, 21). The wide distribution of these receptors across diverse tissues and organs explains the broad range of clinical manifestations observed in COVID-19.

COVID-19 presents a wide respiratory spectrum of symptoms, ranging from mild to severe cases. Common symptoms include fever, dry cough, dyspnea, and fatigue, although asymptomatic infections are widely recognized (22). In addition to respiratory distress, the most prominent symptom of COVID-19 that patients often experience are nausea, vertigo, and headache, which implies the neurological system (18). The pathogenesis of SARS-CoV-2 infection is generally divided into two phases: an initial innate immune response aimed at lung defense, followed by a secondary phase of inflammation-associated tissue injury, which contributes to severe disease outcomes (23). Severe cases can escalate to acute respiratory distress syndrome (ARDS), metabolic imbalances, and multi-organ dysfunction (**Figure 1**).

Several risk factors influence the severity of the disease. Studies indicate that individuals over 65 years old are more likely to develop severe complications and experience higher mortality rates, particularly among men (24, 25). For example, in Italy, older male patients with multiple comorbidities were disproportionately affected (26). In addition to age and sex, socioeconomic disparities and chronic health conditions play a crucial role in disease progression. Patients with preexisting conditions, such as cardiovascular disease, hypertension, diabetes, and metabolic disorders, face an increased risk of developing severe symptoms (27–30). Moreover, behavioral and environmental factors contribute to disease outcomes. Research suggests that men may be more vulnerable to severe COVID-19 complications due to lifestyle habits, occupational exposure, and higher rates of smoking and alcohol consumption (31).

Despite the significant reduction in COVID-19’s global impact due to widespread public health measures and vaccination efforts, the virus remains a concern, with 115,461 cases reported globally in February 2025 (2). The risk of a new SARS-CoV-2 variant or similar viral agents emerging—ones capable of evading vaccines and antivirals—remains a serious concern (32). Although the global response has successfully mitigated much of the pandemic’s impact, the continued circulation of SARS-CoV-2 underscores the need for ongoing vigilance, research, and adaptation of public health strategies.

## 3 Alcohol use and its effects on immune function and mental health

Alcohol consumption, particularly chronic and excessive use, is a well-documented risk factor for both immune system dysfunction and mental health disorders. The interplay between alcohol, immune response, and mental health is complex and involves multiple biological pathways.

The immune system is a complex network of molecules, cells, tissues, and organs that protect the body from infections and pathogens. It consists of two main components: innate and adaptive immunity. The innate immune response is the first line of defense, acting rapidly and non-specifically against pathogens. It relies on immune cells such as natural killer cells, neutrophils, monocytes/macrophages, and dendritic cells, which detect pathogens through pathogen recognition receptors (PRRs), including Toll-like receptors (TLRs) among others. These receptors recognize pathogen-associated molecular patterns (PAMPs), which are common in microorganisms but absent in host cells (33). The immune response within the CNS involves specific cells, primarily microglia that, together with astrocytes and other cells, form the glia (34). They also express TLR mRNAs (35). The activity of TLRs triggers several molecular pathways that lead to the expression of innate immune genes particularly from proinflammatory cytokines and/or interferon (IFN). These chemical messages are critical for initiating innate and adaptive immune responses. PRRs activation also initiates nontranscriptional responses such as the induction of phagocytosis, autophagy, cell death, and cytokine processing (36). Among the genes transcripted are those encoding key pro-inflammatory factors, including tumor necrosis factor alpha (TNF-α), interleukin 1-beta (IL-1β), and interleukin 6 (IL-6), which contribute to an inflammatory state (37). Cytokine release recruits immune cells to clear or contain an immune stimulus, helping to restore homeostasis. In the response against viruses, IFN release plays a crucial role by defending the body from the infections and regulating the immune system. Its primary function is to block viral replication and modulate the immune response, enhancing the body’s ability to control infections (38). Although microglia are primary immune cells, astrocytes influence microglial activation through cytokine release, exacerbating neuroinflammatory cycles (39).

If the innate (general) immune system fails to destroy the pathogens, the adaptive (specialized) immune system takes over. The adaptive immune system specifically targets the type of germ that is causing the infection. This system consists of cell-mediated immunity, driven by T cells, and humoral immunity, led by B cells. CD4-expressing T helper cells are essential for activating and maturing monocytes, cytotoxic T cells (CD8-expressing), and B cells. Cytotoxic T cells target and destroy cancer cells and intracellular pathogens, while B cells differentiate into plasma cells that produce antibodies (immunoglobulins, Ig) to neutralize extracellular pathogens and prevent infection spread (40).

**Figure 2:**
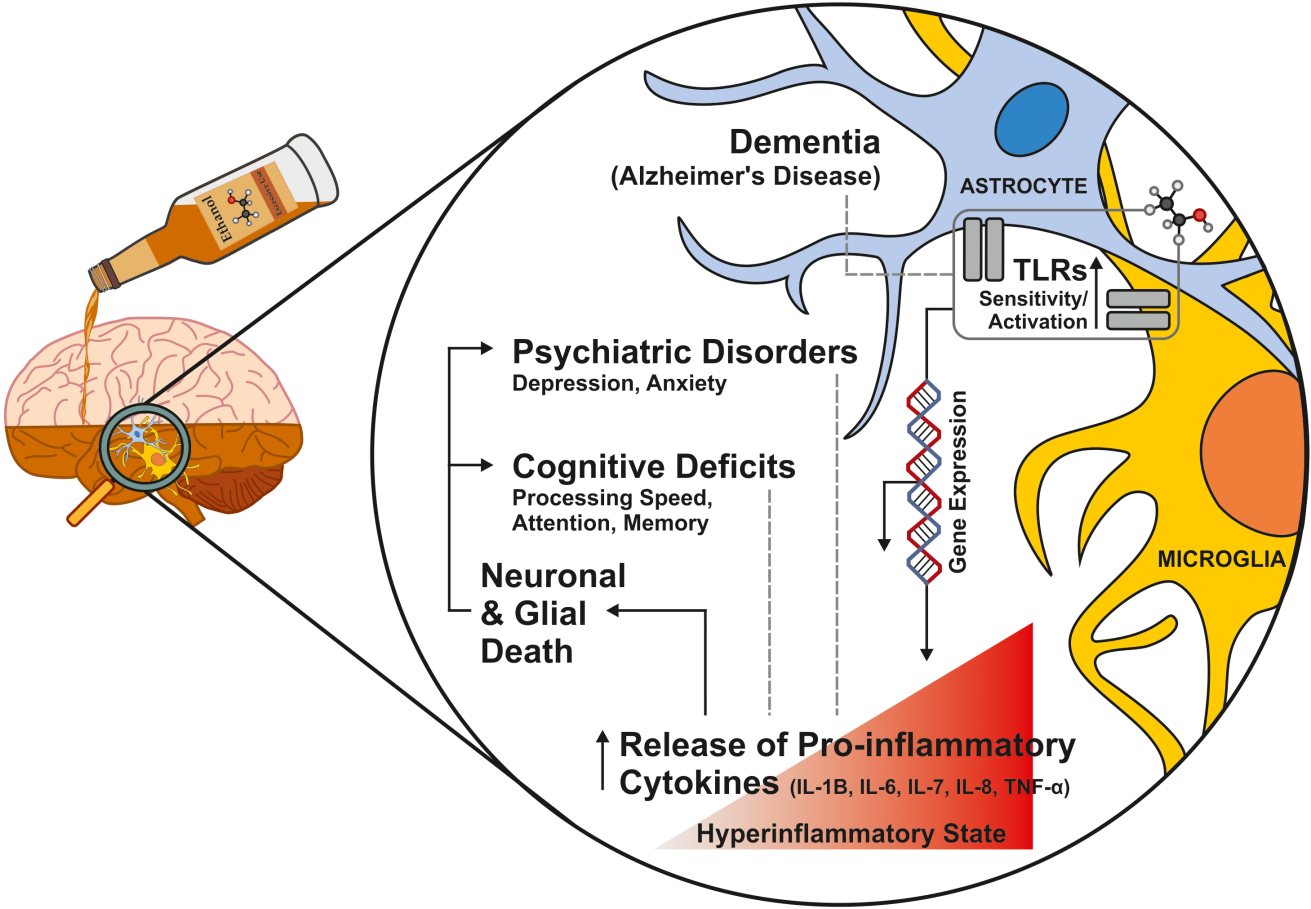
Neuroinflammatory effects of excessive alcohol consumption. Among other inflammatory pathways not addressed in this review, excessive alcohol intake activates and increases the sensitivity of Toll-like receptors (TLRs) expressed in microglia and astrocytes (in particular, TLR4 has been strongly associated with alcohol-induced inflammatory brain damage). Disruption of TLR activity triggers several molecular pathways that lead to a pro-inflammatory environment, partially mediated by the increased expression of pro-inflammatory cytokines. It has been suggested that the pro-inflammatory factors playing a more prominent role in ethanol-induced neuroinflammation are IL-1β, IL-6, IL-7, IL-8, and TNF-α. An enhanced neuroinflammatory response contributes to neuronal loss in regions such as the association frontal cortex, orbitofrontal cortex, hippocampus, limbic system, cerebellum, thalamus, and hypothalamus. Such alterations may help explain the cognitive deficits or the increased risk of developing psychiatric disorders such as anxiety and depression, which exhibit high comorbidity with alcohol use disorder (AUD) and display a pro-inflammatory profile comparable to that elicited by chronic alcohol intake.

Any level of alcohol consumption, whether acute or chronic, exerts immunomodulatory effects. Acute consumption tends to temporarily suppress the immune response, while chronic consumption induces the production of inflammatory mediators, altering the inflammatory response and host defense mechanisms (41, 42). In chronic drinkers, a reduced migration of specific immune system cells is observed, for example, during TLRviral respiratory infections (43) and alterations in inflammatory signaling cascades, resulting in elevated levels of proinflammatory cytokines like TNF-α and IL-6 (44). Chronic alcohol consumption increases the risk and severity of chronic infections with HIV (human immunodeficiency virus) or hepatitis C (42). It is speculated that in this case, alcohol consumption disrupts the necessary cell mechanism responsible for pathogen clearance and impaired antiviral defenses, promoting a pro-inflammatory environment (45). The alteration of the immune system in individuals with alcohol use disorders increases the risk of health complications, including infections, cardiovascular and liver problems (42). Furthermore, chronic alcohol consumption is considered a risk factor for lung diseases (46) and, specifically, for COVID-19 as it is associated with increased disease severity and higher mortality rates (47–49) (**Figure 2**).

Alcohol can cross the BBB, triggering a local pro-inflammatory response in the CNS mediated, in part, by the TLR family. Chronic alcohol consumption is proposed to increase TLR sensitivity, thereby enhancing the expression of pro-inflammatory cytokines (50). Studies in post-mortem brain samples from individuals with AUD have revealed microglial activation alongside a significant increase in pro-inflammatory cytokine production (51, 52). These findings suggest that TLR activation is a key contributor to the neurotoxic effects observed with chronic alcohol exposure. Within the TLR family, several subtypes—namely TLR2, TLR3, TLR4, and TLR7—have been identified as playing crucial roles in neuroinflammation linked to AUD. In particular, TLR4 has been strongly associated with alcohol-induced inflammatory brain damage (53). Recent studies further highlight the critical role of TLR4 and its downstream MyD88 signaling cascade in promoting ethanol-induced neuroinflammation (54) (**Figure 2**).

Although there is still debate over which pro-inflammatory factors are most critical in ethanol-induced neuroinflammation, research indicates that increased levels of IL-1β, IL-6, IL-7, IL-8, and TNF-α play a significant role (55, 56). Additionally, IL-3 and TGF-β1 (transforming growth factor beta 1) also appear to contribute, albeit to a lesser extent (44). The ongoing neuroinflammatory process leads to death of neurons and neuroglial cells in various brain structures, primarily in those associated with the development of a pathological craving for alcohol (57). This process is also associated with mental health problems, such as depression (58) (**Figure 2**).

In addition to alcohol’s ability to activate the local immune system in the CNS, this molecule has a strong effect on the integrity of the BBB. It regulates the expression of structural proteins (zonula occludens-1, VE-cadherin and occludin) and functional proteins (major facilitator superfamily domain-containing protein-2a (Mfsd2a), low-density lipoprotein receptor-related protein-1 (LRP1)), leading to increased permeability in preclinical studies in animal models and human endothelial cells (ECs) (59, 60). The alcohol disruption of the BBB allows circulating inflammatory cytokines to penetrate the brain contributing to local inflammation which can contribute to cognitive decline and mental health issues.

In addition to microglia, astrocytes also play a central role in mediating neuroinflammation induced by alcohol consumption (39). Alcohol exposure activates innate immune receptors on astrocytes, particularly TLR4, and interleukin-1 receptors (IL-1R). This activation triggers downstream signaling cascades involving IRAK, MAP kinases (ERK1/2, p-38, JNK), and transcription factors such as NF-κB and AP-1, leading to the transcription of inflammatory mediators (53, 55).

The relationship between alcohol consumption and immune system activation is bidirectional. Beyond ethanol’s ability to trigger local and systemic immune responses, there is experimental evidence that immune activation mediated by TLRs can enhance motivation to consume alcohol and increase alcohol intake. In animal models, systemic administration of lipopolysaccharides (LPS), which activate innate immune signaling through TLR4, produces prolonged elevations in alcohol consumption, highlighting a strong link between neuroinflammatory processes and alcohol drinking behavior (61). Pharmacological and genetic manipulations of individual TLRs in rodents have been shown to modulate drinking behavior. For instance, knockout of Tlr2 or Tlr3 reduces alcohol consumption in some models (62, 63) whereas chronic TLR3 activation increases drinking (64). TLR7 activation exerts divergent effects on alcohol drinking behavior depending on the temporal pattern of stimulation. Acute TLR7 activation produces a sickness-like response that transiently reduces alcohol intake in rodent models, while repeated administration of TLR7 agonists (e.g., R837, R848) increases voluntary alcohol consumption and operant self-administration (65, 66).

These toxic effects of alcohol on the brain vary depending on the stage of development, dose, pattern of consumption, and duration of exposure (67). Nevertheless, chronic alcohol abuse generally leads to neuronal loss in regions such as the association frontal cortex, orbitofrontal cortex, hippocampus, limbic system, cerebellum, thalamus, and hypothalamus, as well as the connections between these regions (68, 69). These anatomical alterations lead to a wide range of cognitive deficits, including memory impairments, visuospatial processing deficits, attentional dysfunction, poor decision-making, increased impulsivity, and changes in cognitive flexibility and executive functioning overall (69–72). Although some authors suggest that cognitive deficits in patients with AUD are largely underdiagnosed (73), a recent study indicates that such deficits may be present in approximately 43% to 84% of AUD patients (74).

These alcohol-induced cognitive impairments are partly due to a neuroinflammatory imbalance, which disrupts neuronal apoptosis and synaptic function, among other mechanisms (75). Evidence suggests that deficits in domains such as attention, processing speed, and memory are associated with increased levels of proinflammatory markers and decreased levels of anti-inflammatory cytokines. In this regard, pro-inflammatory agents such as IL-6 appear to be a key driver of cognitive decline, whereas the anti-inflammatory cytokine IL-10 may play a protective role against alcohol-induced cognitive impairments (76–78). On the other hand, the deleterious effects of alcohol consumption on declarative memory appear to be partially mediated by proinflammatory components (such as TNF-α) that influence adult neurogenesis by affecting neural stem cell (NSC) proliferation and newborn neuron survival in the hippocampal dentate gyrus. Indeed, NSC proliferation decreases in a concentration-dependent manner following alcohol intoxication in the brain (79). Regarding the impact on cognitive functions, an association has been established between alcohol consumption and the onset of various types of dementia, including Alzheimer’s disease (AD), Parkinson’s disease, Korsakoff syndrome, and vascular dementia. Overall, chronic and excessive alcohol intake constitutes a significant risk factor for these conditions (80), whereas mild to moderate consumption appears to have a protective effect (81). Concerning immune response, studies suggest that AUD and AD share common pathological mechanisms, such as microglial activation and the upregulation of TLR2 and TLR4 (82). However, there is no clear causal relationship between alcohol consumption and AD development via immunological pathways. (**Figure 2**)

Other psychiatric disorders, such as major depressive disorder (MDD) and anxiety-related disorders, exhibit high comorbidity with AUD. Specifically, it has been observed that 40% of MDD patients have experienced AUD at some point in their lives (83), and half of individuals undergoing treatment for problematic alcohol use also meet diagnostic criteria for one or more anxiety disorders (84). Notably, these disorders are characterized by alterations in neuroinflammatory mechanisms (85). For instance, elevated levels of pro-inflammatory markers, including IL-1β, IL-6, TNF-α, and C-reactive protein (CRP), have been reported in various anxiety-related disorders, such as post-traumatic stress disorder, generalized anxiety disorder, and panic disorder (86). Additionally, a meta-analysis of 82 studies including 3,212 participants with MDD found that peripheral levels of IL-6, TNF-α, IL-10, soluble IL-2 receptor, C-C chemokine ligand 2, IL-13, IL-18, IL-12, IL-1 receptor antagonist, and soluble TNF receptor 2 were elevated in MDD patients compared to healthy controls (87). Although several of these markers are also elevated following excessive alcohol consumption, establishing a direct relationship between these inflammatory responses and the aforementioned disorders remains challenging. In fact, not all MDD patients exhibit increased inflammatory markers, and some anti-inflammatory markers are also upregulated in these individuals (87).

## 4 SARS-CoV-2 infection and its impact on immunity and mental health

The immune system plays a critical role in determining the outcomes of SARS-CoV-2 infection, influencing both acute disease severity and long-term health consequences. SARS-CoV-2 infection initiates complex immune interactions involving innate and adaptive responses, which when dysregulated, can lead to extensive tissue and organ damage, contributing to severe clinical manifestations and prolonged symptoms known as Long COVID.

The innate immune response represents the body’s first line of defense against viral infections, including SARS-CoV-2. This response primarily involves antiviral signaling mediated by type I and III IFNs. Upon viral entry, host pattern recognition receptors such as TLRs and retinoic acid-inducible gene-I (RIG-I)-like receptors detect viral RNA and initiate signaling pathways to rapidly produce IFNs. However, SARS-CoV-2 characteristically triggers a delayed and weakened type I IFN response. Specific viral proteins, notably Nsp5 and orf8, disrupt essential signaling molecules, undermining early antiviral defenses, facilitating uncontrolled viral replication, and delaying effective immune activation (88, 89).

This compromised innate immune signaling also promotes excessive inflammatory responses, significantly influencing disease severity. Viral replication in alveolar type II pneumocytes induces pyroptosis, an inflammatory form of programmed cell death. Subsequent release of pathogen-associated and damage-associated molecular patterns (PAMPs and DAMPs) further amplifies immune cell activation, resulting in a hyperinflammatory state or “cytokine storm.” This cytokine storm is characterized by elevated production of pro-inflammatory cytokines, such as IL-1β, IL-6, TNF-α, IP-10, and CCL2, which exacerbate tissue injury, severe lung inflammation, and predispose patients to acute respiratory distress syndrome (ARDS) and systemic organ dysfunction (88–90).

In parallel, SARS-CoV-2 significantly disrupts adaptive immunity involving both B and T lymphocytes. The virus downregulates major histocompatibility complex class I (MHC-I) expression on infected cells, impairing recognition and elimination by cytotoxic CD8+ T cells. Furthermore, SARS-CoV-2 triggers rapid antibody class-switching from IgM to IgG, although this swift shift may not effectively establish lasting immunological memory, potentially weakening long-term immunity. The persistent inflammatory cytokine environment also disrupts adaptive immune cell differentiation and activation, further intensifying immune dysfunction and prolonged viral persistence (88).

The systemic inflammatory response induced by SARS-CoV-2 not only compromises immune regulation but also directly contributes to widespread tissue and organ damage. In the respiratory tract, extensive damage to alveolar type II cells leads to impaired lung function, fibrosis, and respiratory failure. Cardiovascular involvement often manifests as endothelial dysfunction, myocarditis, myocardial fibrosis, and compromised vascular function, significantly raising the risk of cardiac events. Kidney complications frequently include acute kidney injury, tubular and glomerular damage, potentially progressing to chronic renal impairment. Gastrointestinal symptoms commonly result from intestinal inflammation and barrier dysfunction, while pancreatic involvement may disrupt glucose metabolism. Critically, SARS-CoV-2 can invade the CNS, causing significant neuroinflammation characterized by glial activation, neuronal stress, and apoptosis, forming the basis for neurological and cognitive impairments associated with the virus (91).

Emerging evidence indicates that immune dysregulation triggered by SARS-CoV-2 infection can persist beyond the acute phase, potentially driving chronic symptoms known collectively as Long COVID. Persistent changes in immune cell populations, including T-cell exhaustion and disrupted coordination of adaptive responses, contribute to ongoing inflammation observed months after initial infection. These prolonged immune alterations underscore the complex nature of COVID-19 and highlight the importance of deeper mechanistic understanding for developing therapeutic interventions targeting both immediate and long-lasting complications (90, 92).

Long COVID is defined as a chronic condition following SARS-CoV-2 infection, persisting for at least three months and manifesting as continuous, relapsing-remitting, or progressive symptoms affecting multiple organ systems. Despite its recognition, there is still no widely accepted consensus definition among clinicians, researchers, or regulatory agencies, reflecting challenges in clearly delineating this condition (93).

Frequent cognitive and emotional symptoms of Long COVID, such as fatigue, cognitive impairment, anxiety, and mood disorders, have drawn attention to potential direct or indirect viral effects on the CNS. Cranial nerves -including the olfactory, trigeminal, optic, and vagus nerves- and the BBB may serve as key pathways for both direct and indirect interactions between SARS-CoV-2 and the brain. It may disseminate to other brain regions through infected nerve endings, retrograde transport, and transsynaptic transmission (94).

In this context, Long COVID patients have reported new and worsening mental health symptoms, with depression, anxiety, post-traumatic stress disorder (PTSD), and insomnia being the most frequently reported (90). A recent meta-analysis that examined the long-term physical and mental sequelae of COVID-19 found that around 19.7% experienced psychiatric symptoms, with depression (18.3%) and PTSD (17.9%) being the most common. Regarding neurological symptoms, cognitive deficits (19.7%) and memory impairment (17.5%) were reported. Subgroup analysis revealed that individuals at greater risk of long-term sequelae tended to be older, mostly male, residing in high-income countries, and experienced more severe acute infections. Those with severe infection showed higher rates of PTSD, sleep disturbances, cognitive deficits, concentration difficulties, and gustatory dysfunction. Meanwhile, individuals with mild infection exhibited a greater prevalence of anxiety and memory impairment after recovery (95).

Focusing on the affected cognitive domains, another recent meta-analysis found that the greatest impairment was in perceptual motor function, although it did not reach the threshold for a large effect size. When analyzing other studies, the authors reported that executive function was the most frequently assessed cognitive domain, with impairment reported in 69% of cases. Similar rates of impairment were found in learning and memory (82%), perceptual motor function (68%), language (73%), and complex attention (69%). Few studies examined visuospatial and social cognition, but all that did reported some level of impairment in these domains (96). Additionally, “brain fog” is one of the most commonly reported symptoms among millions of individuals with Long COVID. This term describes a cluster of cognitive symptoms, including inattention, short-term memory loss, and reduced mental acuity (97). In a sample of 1680 Long COVID patients, 7.2% reported experiencing brain fog, with higher prevalence observed among women as well as patients with respiratory problems and previous intensive care unit admissions (98).

Various mechanisms have been suggested to account for the nervous system manifestations of COVID-19, such as neuroinflammation, BBB disruption, gut-brain axis dysregulation, along with the emerging vascular disruption hypothesis that emphasizes endothelial dysfunction and hypoperfusion as a key underlying mechanism. In contrast, direct viral neurotropism has less supporting evidence, and no specific anatomical localization has been consistently identified (96, 99). Within the CNS, viral infection can trigger significant neuroinflammation, activating glial cells—particularly astrocytes and microglia—leading to sustained inflammatory signaling. Peripheral cytokine storms further exacerbate this neuroinflammation by compromising the BBB and facilitating immune cell infiltration into the brain. Persistent glial activation and inflammatory responses may thus drive neuronal stress, damage, and neurodegeneration, underpinning cognitive and psychiatric complications observed in Long COVID patients (100–103).

As previously mentioned, the widespread inflammation and tissue damage induced by SARS-CoV-2 may trigger the activation of autoimmune cells, such as memory B cells, playing a role in the persistence of neurological sequelae in Long COVID and the multiorgan involvement characteristic of a dysregulated immune response. The presence of functional autoantibodies in COVID-19 patients has been linked to a range of clinical manifestations, including neurological symptoms (99). On the other hand, SARS-CoV-2 is associated with neuroendothelial dysregulation due to cell death via ACE2 and transmembrane serine receptors (TMPRSS2) on neurovascular endothelial cells, contributing to endothelial dysfunction and neural injury. Viral load and Long COVID severity may contribute to oxidative stress and hypoxia, leading to neuroinflammation, microvascular inflammation, and microthrombi (104).

In addition, astrocytes, which help maintain the BBB, express ACE2 receptors, making them susceptible to viral infection and potential BBB disruption (105). As key regulators of CNS inflammation, microglia may mediate neurological sequelae from cytokine storms, impacting neuronal activity through mechanisms such as heightened astrocyte reactivity, reduced oligodendrocytes, impaired axonal myelination, and decreased hippocampal neurogenesis (106, 107). Thus, several studies such as the one conducted by Peluso and colleagues (108) found increased GFAP, IL-6, MCP-1 and TNF-α in neurologic Long COVID patients. Similarly, findings from Greene and colleagues (109) indicate that brain fog associated with Long COVID is linked to BBB disruption in several brain regions, including the temporal lobes and frontal cortex, as well as to ongoing systemic inflammation. In this study, patients with Long COVID showed elevated levels of IL-8, GFAP, and TGFβ. Notably, TGFβ—previously implicated in the pathogenesis of chronic fatigue syndrome—was particularly elevated in individuals experiencing brain fog.

Recent studies have highlighted the role of pro-inflammatory cytokines such as TNF-α and IL-6 not only in cognitive impairments but also in mood disturbances among COVID-19 survivors. IL-6, in particular, appears to be a key mediator in the link between depression and Long COVID, contributing to immune dysregulation by altering the balance between TH17 and Treg lymphocytes and affecting brain regions associated with mood regulation. Elevated IL-6 levels may also repress brain-derived neurotrophic factor (BDNF), contributing to the development of depressive symptoms (110–112). Additionally, Mazza and colleagues (113) reported that alterations in immune response and systemic inflammation were associated with changes in depressive symptoms over a three-month follow-up period in COVID-19 survivors.

These findings underscore the potential of targeting inflammatory pathways as a therapeutic strategy not only for mitigating the physical severity of COVID-19, but also for addressing its associated mental health symptoms. The inclusion of infliximab—a monoclonal antibody that blocks TNF-α signaling—in clinical trials, supported by its neuroprotective effects in preclinical models (109–111), further highlights the relevance of anti-inflammatory agents within integrated treatment approaches.

## 5 Synergistic effects of alcohol use disorder and SARS-CoV-2: clinical, immunological, and neuropsychiatric implications

Severe alcohol consumption and SARS-CoV-2 infection each pose significant threats to immune competence and mental health. When they co-occur, their effects may not be merely additive but synergistic, leading to compounded health risks. As stated earlier, chronic alcohol consumption modulates the immune system in a dysregulated manner—suppressing certain responses while promoting chronic inflammation—which increases vulnerability to infections, particularly of the respiratory tract, such as pneumonia and influenza. These alterations weaken mucosal barriers and antiviral defenses, creating a biological environment that facilitates the entry and progression of pathogens like SARS-CoV-2. Similarly, COVID-19 can range from mild symptoms to severe respiratory distress, systemic inflammation, and long-term complications, with severity influenced by pre-existing conditions.

Substance use disorders—including heavy smoking, opioid use, and alcohol consumption—have been consistently identified as risk factors for adverse COVID-19 outcomes (24, 114, 115). Evidence from a prospective cohort study by Kianersi and colleagues (116), conducted among U.S. college students, showed that high-risk alcohol consumption (AUDIT ≥ 8) was associated with a 2.44-fold increase in SARS-CoV-2 seroconversion (RR = 2.44, 95% CI = 1.35–4.25) and a 1.84-fold higher likelihood of self-reported infection (RR = 1.84, 95% CI = 1.04–3.28), compared to students with lower-risk drinking patterns. These associations remained significant after adjusting for demographic and behavioral variables. Although no statistically significant difference was found in the incidence of symptomatic COVID-19, the study supports the notion that alcohol use may increase susceptibility to infection, even in younger populations with low comorbidity burden.

Building on these findings, a comprehensive meta-analysis by Wei and colleagues (117), which included over 1.6 million individuals with COVID-19, provided robust evidence linking alcohol consumption with poor clinical outcomes. The authors found that people with any alcohol use history had a 23% increased risk of severe or critical COVID-19 (RR = 1.23, 95% CI = 1.02–1.48), a 79% higher risk of hospitalization (RR = 1.79, 95% CI = 1.75–1.82), and a 32% higher risk of ICU admission (RR = 1.32, 95% CI = 1.08–1.60). Notably, excessive drinkers showed a 125% higher risk of hospitalization compared to never drinkers. These findings highlight the clinical relevance of alcohol consumption, particularly at high levels, in shaping COVID-19 prognosis through its effects on immune function, inflammation, and organ vulnerability.

### 5.1 Shared biological mechanisms between chronic alcohol use and SARS-CoV-2 infection

The synergistic health risks observed in individuals with both alcohol use disorder and SARS-CoV-2 infection stem from overlapping and mutually reinforcing biological mechanisms. Chronic alcohol consumption and COVID-19 independently disrupt immune regulation, damage key organ systems, and promote systemic inflammation. When combined, these effects may lead to amplified pathophysiological responses.

At the immune level, both conditions interfere with host defense by altering the balance between pro- and anti-inflammatory pathways. Chronic alcohol use impairs the function of innate immune components such as alveolar macrophages, neutrophils, and natural killer cells, while also reducing levels of antimicrobial peptides like LL-37 (118). Furthermore, alcohol compromises pulmonary barrier integrity by lowering surfactant protein D (SP-D) (119) and secretory IgA (sIgA) (120), weakening mucosal immunity and facilitating viral entry. Similar alterations have been observed in SARS-CoV-2 infection, which also dysregulates cytokine responses and epithelial integrity, often leading to hyperinflammation and multi-organ damage.

Also, SARS-CoV-2 infection robustly activates TLR4 and TLR7 pathways, leading to peripheral and central neuroinflammation, microglial activation, and BBB disruption (121, 122). Experimental models show that the SARS-CoV-2 spike protein induces TLR4-dependent microglial activation and synaptic loss, effects prevented by genetic or pharmacological inhibition of this pathway (122). Although direct evidence linking COVID-19-related TLR activation to increased alcohol craving, relapse, or withdrawal severity is not yet available, both alcohol- and infection-driven immune responses converge on neuroimmune circuits involved in stress responsivity and reward regulation. These shared mechanisms may suggest that SARS-CoV-2–induced immune priming could exacerbate craving and relapse vulnerability in individuals with alcohol use disorder, a hypothesis that warrants further empirical investigation.

One particularly relevant point of convergence lies in the modulation of ACE2, the primary entry receptor for SARS-CoV-2. Friske and Spanagel (123) report that chronic alcohol consumption increases ACE2 expression in tissues such as the lung, liver, and brain—potentially enhancing susceptibility to viral invasion. In parallel, both alcohol and COVID-19 disrupt gut barrier function and promote the translocation of microbial products like LPS, activating systemic inflammatory cascades via Toll-like receptors (e.g., TLR4) and MyD88 signaling (124). This shared disruption of the gut–liver–lung axis may contribute to the heightened and prolonged immune activation observed in individuals with both conditions.

Expanding on these mechanistic links, recent experimental evidence has demonstrated a direct synergistic interaction between ethanol exposure and the SARS-CoV-2 spike protein itself. In K18-hACE2 mice, chronic alcohol intake upregulated pulmonary ACE2 expression and markedly exacerbated S1 spike–induced lung injury and inflammation, suggesting that alcohol potentiates spike-mediated tissue damage and respiratory dysfunction (125).

Additionally, chronic alcohol use and SARS-CoV-2 infection affect overlapping organ systems, particularly the lungs, liver, cardiovascular system, and CNS. Alcohol-related impairments in these organs may reduce resilience to SARS-CoV-2–induced injury, while COVID-19 may further aggravate pre-existing damage. In the brain, both agents promote neuroinflammation, BBB dysfunction, and glial activation—mechanisms implicated in cognitive and emotional disturbances (126, 127). All together, these findings suggest that chronic alcohol use does not merely coexist with SARS-CoV-2 infection as a risk factor—it amplifies the biological vulnerabilities that drive disease progression and possibly contributes to persistent post-infectious complications.

In this context, smoking deserves particular consideration as a highly prevalent comorbidity among individuals with AUD that may further exacerbate respiratory and immune vulnerability. Chronic smoking upregulates ACE2 and transmembrane serine protease 2 (TMPRSS2) expression in the respiratory epithelium, facilitating SARS-CoV-2 entry and replication (128). Both tobacco and alcohol impair mucociliary clearance, damage alveolar macrophages, and enhance oxidative stress, thereby compromising pulmonary defense (129). These overlapping effects increase the risk of severe COVID-19, hospitalization, and acute respiratory distress syndrome, particularly in individuals with dual exposure. Moreover, smoking has been identified as a risk factor for Long COVID, possibly through persistent lung inflammation and immune dysregulation (129). Given the high co-occurrence of smoking and AUD, this combination likely produces additive or synergistic effects on pulmonary and immune dysfunction, amplifying susceptibility to both severe and prolonged COVID-19 outcomes.

Together, the combined effects of alcohol and smoking on respiratory and immune integrity not only heighten susceptibility to infection but may also alter the efficacy of immune responses, including those induced by vaccination. Chronic alcohol consumption may interfere with the immune response to vaccines, including those against SARS-CoV-2. Animal studies suggest that the effects depend on timing and dose: in mice, alcohol intake prior to Bacille Calmette-Guérin (BCG) vaccination impaired adaptive immunity and control of infection, while post-vaccination drinking did not (130). Similarly, rhesus macaques exposed to high blood ethanol concentrations (BEC > 80 mg/dL) showed suppressed T- and B-cell responses to Modified Vaccinia Ankara (MVA) vaccination, whereas moderate drinking (BEC < 50 mg/dL) enhanced immune responses (41). These effects were accompanied by transcriptional and epigenetic changes in immune-related pathways.

Although direct evidence from human studies on COVID-19 vaccine responses in individuals with alcohol use disorders remains limited, emerging data point to a negative association between alcohol consumption and vaccine-induced immunogenicity. A large observational study among over 3,000 healthcare workers in Japan found that even moderate alcohol intake was significantly associated with reduced anti-SARS-CoV-2 spike IgG antibody titers following administration of the BNT162b2 mRNA vaccine. In this study, alcohol consumption was assessed as a regular drinking pattern rather than intake on the day of vaccination, and participants were classified according to their average daily consumption (from <1 go to ≥2 go per day, where 1 go ≈ 23 g ethanol). Thus, the observed effect reflects habitual drinking behavior rather than acute exposure at vaccination The decline in immunogenic response was dose-dependent, with the most marked reduction observed at low-to-moderate levels of alcohol consumption (131).

However, more recent longitudinal cohort evidence suggests that these associations may not generalize across populations. In a study covering up to four vaccine doses (132), found that alcohol use, when classified according to national guidelines, did not predict anti-S or anti-R IgG/IgA/IgM levels, neutralizing activity, or antibody decay rates after vaccination. These findings suggest that while ethanol can potentiate spike-related tissue injury, vaccine-elicited humoral responses may remain largely intact at the population level.

Together, the available data indicate that alcohol-induced immune dysregulation may increase vulnerability to infection and to spike-mediated tissue injury, while its impact on vaccine-induced protection appears to depend on dose, timing, and chronicity. Further research should clarify these interactions, particularly among individuals with heavy or prolonged alcohol use.

### 5.2 Alcohol use, neuroinflammation, and Long COVID (cognitive and emotional symptoms)

Building on these concerns, the persistent consequences of SARS-CoV-2 infection—collectively known as Long COVID—have become an increasing focus of clinical and scientific attention. These include a range of neuropsychiatric symptoms such as fatigue, cognitive impairment, depression, anxiety, and sleep disturbances, which can persist for weeks or months beyond the acute phase. Although the precise mechanisms underlying Long COVID are not yet fully elucidated, current evidence consistently implicates sustained immune activation, neuroinflammation, endothelial dysfunction, and alterations in the BBB.

**Figure 3:**
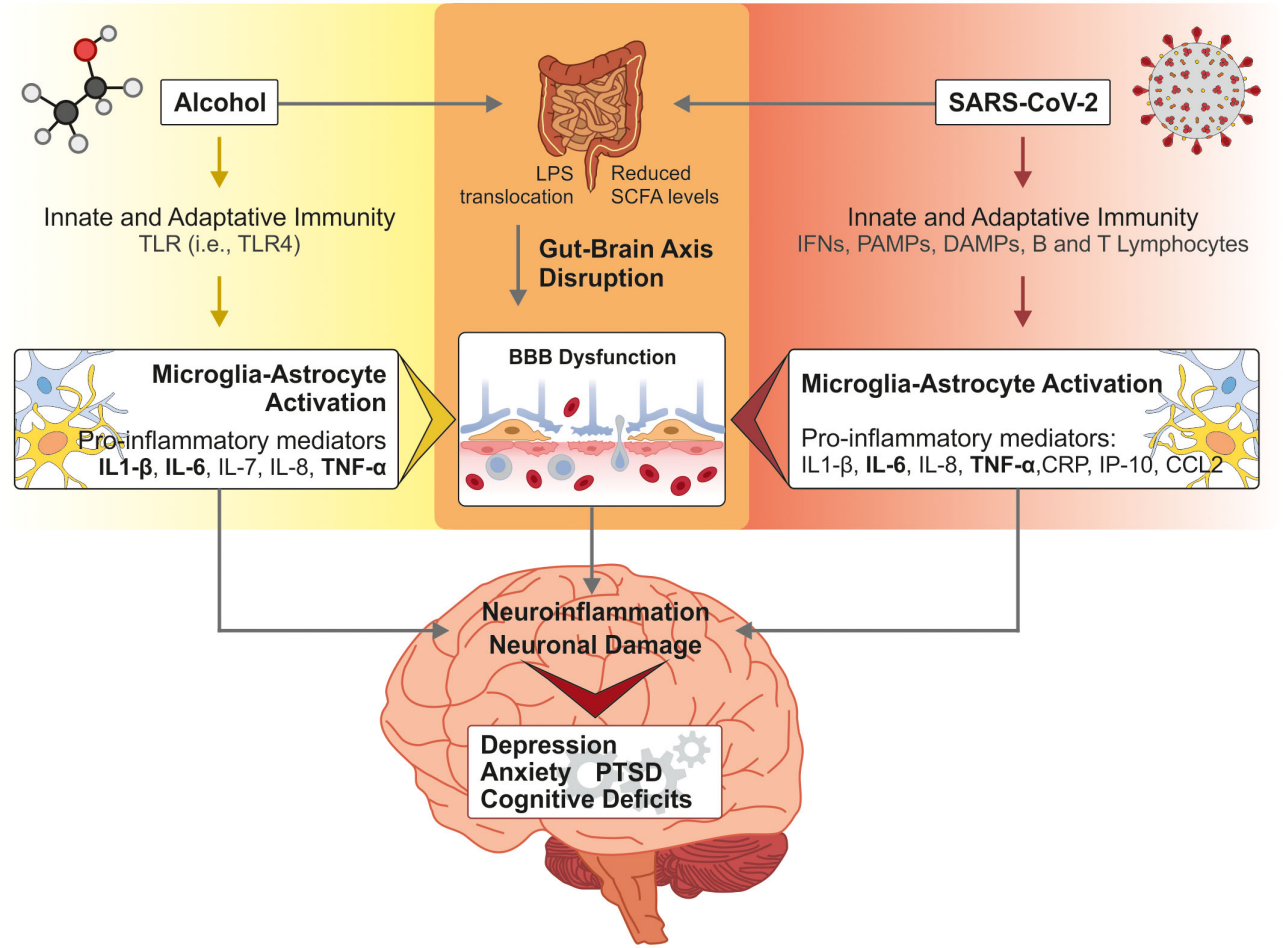
Shared Biological Mechanisms Between Chronic Alcohol Use and SARS-CoV-2 Infection. Both excessive alcohol consumption and SARS-CoV-2 infection alter innate and adaptive immune responses, leading to a pro-inflammatory state through the activation of microglia and astrocytes. This enhanced neuroinflammatory state contributes to neuronal damage, which can lead to cognitive deficits or the increased risk of developing psychiatric disorders. In parallel, both alcohol and SARS-CoV-2 disrupt gut barrier function and promote the translocation of microbial products like lipopolysaccharide (LPS), activating systemic inflammatory cascades via Toll-like receptors. Furthermore, in the brain, both agents promote blood–brain barrier (BBB) dysfunction, mechanism implicated in cognitive and emotional disturbances. These findings suggest that chronic alcohol use does not merely coexist with SARS-CoV-2 infection as a risk factor, it amplifies the biological vulnerabilities that drive disease progression and possibly contributes to persistent post-infectious complications.

Chronic alcohol use is known to induce neuropathological changes that closely mirror those implicated in Long COVID. Studies have shown that repeated ethanol exposure leads to glial activation, increased expression of central pro-inflammatory cytokines (e.g., IL-1β, TNF-α), and BBB dysfunction (133). These effects can persist even during periods of abstinence and are associated with cognitive deficits and affective disturbances frequently observed in both AUD and post-COVID conditions (**Figure 3**).

Building on this evidence, Muhammad and colleagues (49). propose that chronic alcohol use may sensitize the brain’s immune system, making it more reactive to subsequent inflammatory challenges such as SARS-CoV-2 infection. This process—referred to as neuroimmune “priming”—describes a state in which microglial cells and other central immune components become dysregulated due to long-term alcohol exposure, amplifying the inflammatory response when a secondary insult occurs. Muhammad and colleagues (49) highlight that both alcohol and SARS-CoV-2 independently disrupt the BBB, increase oxidative stress, and activate the hypothalamic–pituitary–adrenal (HPA) axis, creating a shared biological substrate for persistent neuroinflammation. These converging mechanisms may contribute to more severe or prolonged symptoms such as cognitive impairment, mood disturbances, and anxiety in individuals with a history of alcohol misuse. Importantly, such cognitive and emotional alterations may, in turn, exacerbate maladaptive drinking patterns, intensify craving, and complicate withdrawal processes in individuals with alcohol use disorder, thereby reinforcing the neuroimmune and behavioral cycle of alcohol-related vulnerability. Beyond these direct neuroimmune interactions, the prolonged systemic and neuropsychiatric effects of SARS-CoV-2 infection raise additional concerns regarding vulnerability to alcohol misuse and relapse. Emerging evidence suggests that individuals experiencing Long COVID, often present depression, anxiety, fatigue, cognitive dysfunction, and sleep disturbance, symptoms strongly associated with increased alcohol consumption and relapse risk (134). Mechanistic and clinical studies further indicate that both COVID-19 and chronic alcohol use disrupt HPA axis regulation and promote neuroinflammation, processes known to heighten stress responsivity and craving (135). Recent findings among U.S. veterans also show that individuals living with Long COVID report poorer sleep quality and higher levels of alcohol use compared to controls without post-COVID symptoms (136). Although direct longitudinal data remain limited, these observations support a plausible hypothesis that the neuropsychiatric sequelae and chronic stress associated with Long COVID may act as triggers or amplifiers of alcohol craving and relapse vulnerability, especially in those with a history of AUD.

Beyond the direct neuroimmune mechanisms discussed earlier, increasing evidence highlights the role of the microbiota–gut–brain axis (MGBA) in the development and persistence of neuropsychiatric symptoms following SARS-CoV-2 infection. This bidirectional communication system regulates immune responses, BBB integrity, and neural signaling. Disruptions in the MGBA—through intestinal dysbiosis and increased gut permeability—can trigger systemic inflammation and neuroinflammatory responses, contributing to symptoms such as fatigue, cognitive impairment, and mood disturbances (124, 137).

Both chronic alcohol use and SARS-CoV-2 infection have been independently associated with gut dysbiosis, reduced abundance of beneficial microbial species, and compromised intestinal barrier function (138). These alterations facilitate the translocation of bacterial endotoxins like LPS into circulation, which in turn activate central immune pathways and disrupt BBB integrity (137, 139). When co-occurring, alcohol and COVID-19 may act synergistically to intensify inflammatory signaling along the gut–brain axis and hinder neurological recovery (**Figure 3**).

A key component in this process seems to be the depletion of short-chain fatty acid (SCFA)–producing bacteria, particularly those that synthesize butyrate—a metabolite essential for maintaining epithelial barrier integrity and modulating inflammatory responses in both the periphery and the brain (137, 139). Reduced SCFA levels have been linked to increased neuroinflammation and behavioral symptoms such as depression and cognitive dysfunction in both experimental models and clinical populations (140).

Moreover, Chen and Vitetta (141) have proposed that gut dysbiosis may help sustain the “brain cytokine storm” observed in COVID-19-related neurological complications, particularly in individuals with pre-existing microbiota alterations such as those seen in alcohol use disorder. In this context, alcohol-induced disruptions of the MGBA may serve as a biological amplifier of Long COVID symptoms.

Taken together, these findings suggest that alcohol-related alterations in the gut–brain axis may exacerbate post-COVID neuropsychiatric outcomes by prolonging systemic and central inflammation. Therapeutic strategies aimed at restoring microbial balance—such as probiotics, prebiotics, dietary changes, or SCFA supplementation—deserve further exploration, particularly in patients with a history of alcohol misuse.

More broadly, the evidence reviewed throughout this section supports the notion that chronic alcohol consumption not only increases susceptibility to SARS-CoV-2 infection and worsens acute outcomes, but also amplifies the biological mechanisms that underlie long-term complications. Through shared pathways of immune dysregulation, organ vulnerability, neuroinflammation, and disruption of the MGBA, alcohol may act as a catalyst for more severe and persistent neuropsychiatric symptoms in the context of COVID-19. These overlapping effects are not merely additive but potentially synergistic, increasing the burden of post-COVID sequelae in individuals with alcohol use disorder.

Beyond these biological mechanisms, pandemic-related anxiety and social stressors have also contributed to marked shifts in alcohol use patterns and sales worldwide. While there is no direct evidence linking anxiety about the newly developed COVID-19 vaccines to changes in liquor sales, several studies indicate that general pandemic-related anxiety was associated with increased alcohol consumption, particularly among women (142) reported that higher coronavirus-related anxiety scores significantly predicted increased drinking frequency and earlier-day consumption, whereas Castaldelli-Maia et al. (143) documented a 20% rise in retail liquor sales in the United States during early pandemic months, interpreted as stress-driven home drinking. These findings suggest that broader psychosocial distress—rather than vaccine-specific anxiety—was the main driver behind the observed increases in alcohol use during the pandemic.

In addition to the clinical aspects addressed throughout this paper, the available evidence highlights important public health implications. Several studies reported significant changes in alcohol use during the COVID-19 pandemic, although these were not uniform across regions or populations. While overall consumption declined in some areas—partly due to restrictions on social gatherings and reduced alcohol availability—other groups, particularly individuals with pre-existing alcohol use disorders or increased psychological distress, experienced a rise in both frequency and quantity of alcohol consumption (144–146). This trend toward solitary and home-based drinking was especially pronounced in countries like the United States and the United Kingdom. In contrast, some European nations reported reductions in heavy episodic drinking, attributed to the closure of bars and limitations on nightlife (147). In Italy, emergency departments recorded a post-lockdown increase in alcohol intoxication cases among adolescents and young adults (148), while projections from England estimate substantial increases in alcohol-related diseases and healthcare burden if current drinking patterns persist (149).

Since the official end of the pandemic in 2023, alcohol consumption has only partially returned to pre-pandemic levels. The rise of online sales and off-premise consumption, economic uncertainty, and evolving social norms have contributed to a lasting shift in drinking behaviors. At the same time, movements such as “sober curious” have gained momentum, particularly among younger adults, reflecting a complex post-pandemic landscape (150, 151). However, several studies indicate that the prevalence and clinical severity of AUD increased during the pandemic and have remained elevated thereafter, particularly among individuals with pre-existing mental health conditions or dual disorders (152, 153). These findings suggest that, although overall consumption has stabilized or declined in some regions, the burden of AUD and its related complications may persist beyond the pandemic. This highlights the importance of sustained, evidence-based public health strategies aimed at monitoring long-term trends and addressing alcohol-related harm among vulnerable populations. The pandemic has exposed certain weaknesses in public health systems and highlighted the need for more resilient and integrated responses to health crises. At the same time, it has underscored the value of strong healthcare infrastructure, a well-trained workforce, universal access to care, and robust surveillance systems, which have proven to be key factors in reducing COVID-19 mortality (154, 155).

Given the significant impact of the pandemic on vulnerable populations—particularly those with substance use disorders—and the established influence of alcohol consumption on COVID-19 outcomes, addressing this issue requires a comprehensive and coordinated public health strategy. As highlighted by Card-Gowers and colleagues (149), a coherent strategy aimed at reducing alcohol harm, preventing further widening of inequalities, and alleviating pressure on the healthcare system will be crucial in mitigating these long-term consequences.
